# Evaluating the pivotal role of MRI in craniocervical junction injury diagnosis: A case report

**DOI:** 10.1097/MD.0000000000042154

**Published:** 2025-05-23

**Authors:** Mahyar Daskareh, Saeid Esmaeilian, Elham Rahmanipour, Mohammad Ghorbani

**Affiliations:** a Department of Radiology, Ziaeian Hospital, Tehran University of Medical Sciences, Tehran, Iran; b Department of Radiology, Shiraz University of Medical Sciences, Shiraz, Fars, Iran; c Immunology Research Center, Mashhad University of Medical Sciences, Mashhad, Iran; d Department of Orthopaedics Surgery, Orthopedic Research Center, Mashhad University of Medical Sciences, Mashhad, Iran.

**Keywords:** apical ligament, craniocervical junction, magnetic resonance imaging, spine trauma

## Abstract

**Rationale:**

Diagnosing craniocervical junction (CCJ) traumatic injuries at initial evaluation is challenging due to patient noncooperation, pain, and multiple traumas, often leading to missed diagnoses with long-term consequences.

**Patient concerns:**

A 35-year-old male with chronic neck pain and neurological symptoms caused by an undiagnosed CCJ injury from a childhood motor vehicle accident.

**Diagnoses:**

The initial radiographs showed normal atlanto-dental interval, basion-dense interval, and basion-axial interval measurements. Symptoms prompted a magnetic resonance imaging (MRI), which revealed a missed hematoma deep to the tectorial membrane, exerting pressure on the cervical cord, along with ligamentous injuries, confirming chronic compression causing myelomalacia and cervical cord atrophy, and atlantoaxial instability from a ruptured apical ligament.

**Interventions:**

Over 5 years of conservative treatment (physical therapy, pain management) failed to relieve symptoms. Post-MRI, management shifted to neurosurgical and orthopedic consultations, with consideration of surgical stabilization.

**Outcomes:**

Prolonged conservative treatment was ineffective due to undiagnosed injuries, resulting in persistent symptoms and neurological deficits. Delayed MRI diagnosis limited outcomes, with management focused on stabilizing the CCJ to prevent further deterioration.

**Lessons:**

Radiographs and computed tomography are limited in detecting CCJ soft tissue injuries. MRI is essential for identifying hematomas and ligament damage in high-velocity trauma, enabling timely intervention to prevent long-term neurological complications.

## 1. Introduction

Diagnosing craniocervical junction (CCJ) traumatic injuries at initial evaluation is frequently challenging due to patient noncooperation, pain, and multiple traumas.^[1]^ Critical structures of the CCJ, such as the tectorial membrane (TM), transverse atlantal ligament (TAL), and apical ligaments, are essential for cervical stability.^[2]^ These structures should be meticulously evaluated using high soft tissue resolution magnetic resonance imaging (MRI).^[3]^

In an acute trauma setting, stability evaluation is often based on craniometric measurements derived from radiographs or computed tomography (CT) imaging. Common radiological measures include the anterior atlanto-dental interval (ADI), basion-dense interval (BDI), and basion-axial interval (BAI).^[3]^ However, these measurements can vary based on the modality and position of the cervical spine, and dynamic changes can affect the visualized bone structure.^[4–6]^

Despite normal initial radiographic findings, injuries to CCJ ligaments can result in significant instability and neurological deficits if not accurately diagnosed.^[2,7,8]^ This case report illustrates the limitations of radiographs and CT in detecting complex CCJ injuries and highlights the critical role of MRI in identifying soft tissue and ligamentous damage, ultimately influencing patient management and outcomes. By focusing on the necessity of advanced imaging, particularly MRI, this case underscores the importance of timely and accurate diagnosis to prevent long-term complications and improve therapeutic interventions for patients with high-velocity cervical trauma.

## 2. Case presentation

A male patient, aged 35, presented with ongoing, long-lasting nonradicular neck discomfort. On the physical examination, his upper extremity strength was recorded as 4/5, with no sensory deficits. His medical history included a motor vehicle accident (MVA) at the age of 16. At the time of the injury, his Glasgow Coma Scale score was 14 out of 15. He had normal findings on physical examination, anteroposterior and lateral radiographs, and CT scan with normal ADI, BDI, and BAI. There were no reported congenital anomalies or significant family medical history.

The patient underwent extensive conservative treatment for more than 5 years. His symptoms included fatigue, nonspecific neck pain, muscular weakness in the upper and lower extremities, as well as paresthesia in the arms and legs. Blood work showed the following results: Hb: 15.6 g/dL, WBC: 8500, vitamin D level: 29 ng/mL, vitamin B12 within the standard range, calcium: 8.9 mg/dL, and magnesium: 1.9 mg/dL. On physical examination, reduced sensation to light touch, distal muscle weakness in the upper and lower extremities, and mild stiffness in both legs were noted. Neck examination revealed tenderness and stiffness in the paraspinal muscles. Exaggerated deep tendon reflexes were observed in the knee and ankle jerks. There were no signs of bowel or bladder dysfunction, gait abnormalities, or dysarthria.

**Figure 1.**
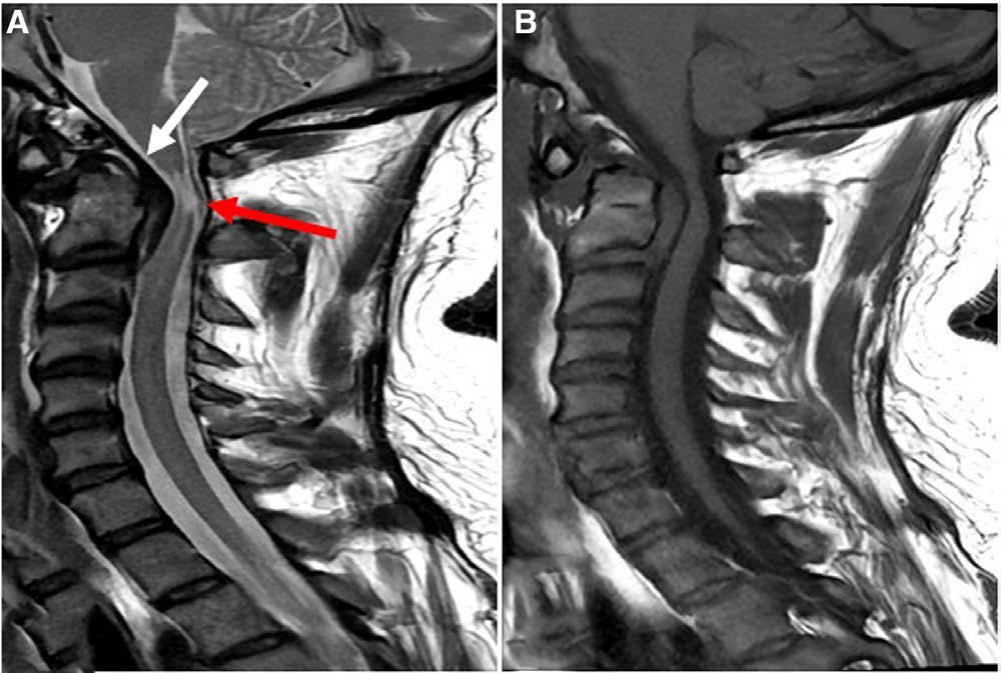
Imaging findings of a missed pretectal membrane hematoma in a 36-year-old male individual. Sagittal T2-weighted image (A) and sagittal T1-weighted image (B) fast spin echo show spinal cord myelomalacia (red arrow) due to a large old hematoma located deep to the tectorial membrane (white arrow). The hematoma demonstrates mildly hyperintense internal content with a peripheral low signal border on the T2-weighted image and iso-signal appearance on the T1-weighted image relative to the cord’s white matter.

Due to his recurrent nonspecific cervical pain and continued mild paresthesia in the arms and legs, despite conservative treatments, a cervical MRI was ordered and performed on a 1.5 Tesla scanner. The protocol included sagittal T1-weighted image, T2-weighted image, and short tau inversion recovery sequences, in addition to axial gradient recalled echo sequence with a slice thickness of 4 mm. Several critical findings were identified on imaging. An old hematoma deep to the TM was noted, exerting pressure on the cervical cord (Fig. 1B). The hematoma was located deep to the TM and superficial to the transverse ligament (Fig. 1A). This chronic compression had led to myelomalacia and cervical cord atrophy. The TM itself appeared intact (Fig. 1A).

**Figure 2.**
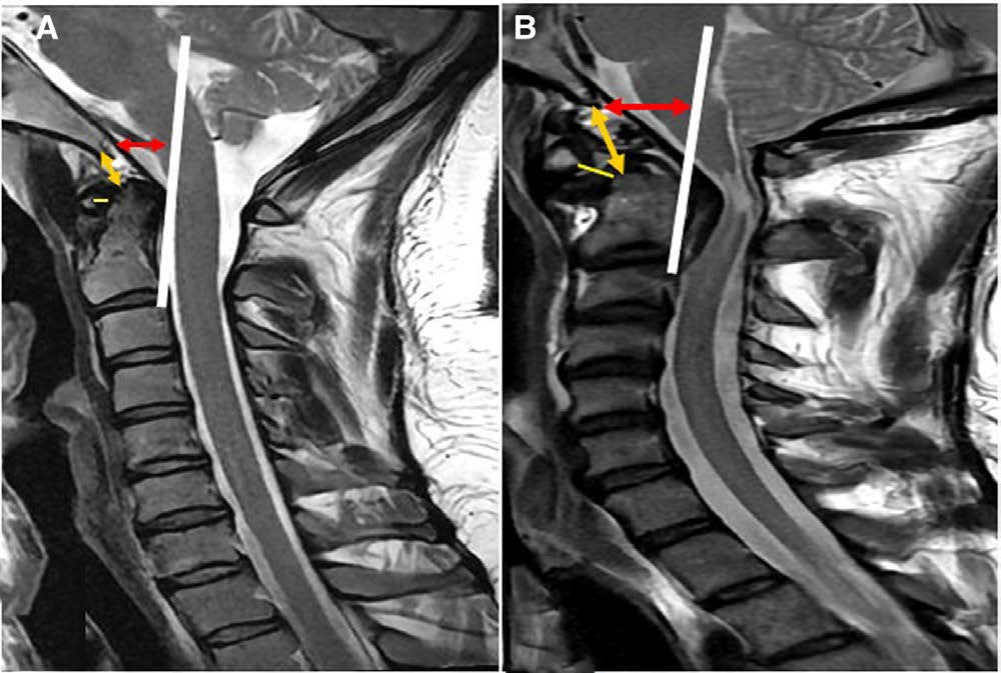
CCJ measurements: ADI (yellow line), BDI (orange line), and BAI (double-headed red arrow) in the sagittal T2-weighted MRI. (A) Sagittal T2-weighted MRI of a companion subject showing normal CCJ Indices. (B) Sagittal T2-weighted MRI of the presented patient with an old missed cervical spine injury demonstrating abnormal measurements (ADI = 6 mm, BDI = 18 mm, and BAI = 15 mm). ADI = atlanto-dental interval, BAI = basion-axial interval, BDI = basion-dense interval, CCJ = craniocervical junction, MRI = magnetic resonance imaging.

A rupture of the apical ligament and intact TM were suggested by an increased BDI (Fig. 2B). Additionally, an increased BAI (Fig. 2B) confirmed complex atlantoaxial instability, and the ADI also increased (Fig. 2B). Since absence of any abnormalities of these indices detected in the CT scan and radiographs interpretation at the time of trauma and considering the painful neck movement without tenderness in patient, we decided against ordering an MRI. Therefore, it might be related to an initially overlooked partial ligament injury in the CCJ, which later developed into a complete ligament injury during the healing process. Moreover, this resulted in an inability to maintain proper alignment in the craniocervical region, along with the impact of other ligament injuries that caused instability and subsequent outcomes.

## 3. Discussion

Literature provides extensive evidence on the complex nature of CCJ injuries, underscoring the crucial role of advanced imaging modalities in their diagnosis and management. This review highlights the inherent limitations of radiographs and CT scans in identifying such injuries. While MRI offers superior sensitivity and specificity in evaluating the soft tissues and ligaments of the CCJ, making it indispensable in high-velocity cervical trauma scenarios.

The case described in this study demonstrates that the childhood MVA led to an undiagnosed multi-ligament injury involving multiple ligaments of the CCJ and a deeply located hematoma. Over time, this hematoma, along with the associated ligamentous ruptures, exerted sustained pressure on the corticomedullary junction, leading to myelomalacia. Literature suggests that radiologic and imaging illustrations of missed hematomas are often inadequately depicted.^[9]^ This study underscores the importance of using effective imaging modalities to promptly detect severe cervical cord injuries, preventing irreversible neurological damage.

Radiographs are commonly used in the initial assessment of cervical spine injuries but have significant limitations in sensitivity and specificity for detecting complex ligamentous injuries and hematomas. Flexion/extension radiographs are particularly suboptimal for evaluating obtunded trauma patients with suspected ligamentous injury.^[10–12]^ Due to their limited ability to reveal soft tissue injuries and subtle bony abnormalities, reliance on radiographs alone can lead to missed diagnoses and delayed treatment.^[13]^

CT imaging has improved sensitivity over plain radiographs and provides better detail of bony structures, making it preferred in many trauma settings.^[4]^ The accuracy of radiologic indices and metrics for initial trauma evaluations has been extensively studied, highlighting their advantages and limitations.^[13,14]^ Fiester et al showed that an anterior ADI of more than 2 mm is not a valid method for screening trauma patients for TAL injuries and AAI. They found that just 11% patients with verified TAL impairment damage had an aberrant ADI of 3 mm or larger.^[15]^ BAI measurement on CT scans is also unreliable due to large inter-examiner variability.^[10,15]^ This emphasizes the significance of cervical MRI in directly assessing the TAL during the diagnostic evaluation and management of individuals with acute high-velocity cervical spine injuries.^[15]^

MRI is considered the best modality for detecting cervical spine injuries, particularly those involving soft tissues and ligaments.^[9,16]^ MRI provides superior sensitivity for identifying ligamentous injuries, hematomas, and other soft tissue damage.^[17]^ Patients who have experienced a severe injury and have an unreliable neurological evaluation should have an MRI of the cervical spine, even if there is no indication of abnormalities in the CCJ on a CT scan.^[18]^ While the clinical usefulness and cost-effectiveness of this approach should be assessed in future trials, the significant expense associated with overlooking a CCJ injury supports the use of MRI with a lower threshold.^[19]^

Research has shown that MRI is more sensitive than CT or plain radiographs alone in identifying cervical spine injuries. Furthermore, including MRI into CT imaging can lead to changes in how patients are treated.^[8,19,20]^ Early detection of ligamentous injuries and associated hematomas on MRI can prompt timely interventions, such as surgical drainage, to prevent long-term complications like cord atrophy.^[21]^

According to recent original studies, MRI should be obtained in patients presenting with Glasgow Coma Scale of 13,^[22]^ persistent neck pain,^[23]^ and hyper-reflexia^[24]^ on neurologic examinations of extremities to ensure comprehensive evaluation of potential ligamentous and soft tissue injuries. Our literature review highlights several studies that demonstrate the superior sensitivity of MRI in detecting such injuries, which are often missed on radiographs and CT scans.

Based on extensive literature review, several clinical symptoms, signs, and trauma mechanisms warrant the use of CCJ-MRI.^[25]^ These include persistent neck pain or radiculopathy unresponsive to conservative treatment, myelopathy symptoms such as limb weakness, numbness, tingling, and exaggerated deep tendon reflexes, high-energy mechanisms of trauma such as MVA which may cause complex ligamentous injuries, neurological red flags including progressive neurological deficits, gait abnormalities, or bladder/bowel dysfunction, and co-existing injuries such as vertebral fractures, head injuries, or other major traumas that may mask underlying CCJ injuries.^[1,7]^

The indications for CCJ-MRI outlined in Table 1 are necessary for comprehensive evaluation and management of patients with suspected CCJ injuries. This table serves as a guideline for clinicians to identify cases where MRI can detect serious injuries that might otherwise be missed, facilitating early intervention and better outcomes.

**Table 1:** Suspected CCJ injury magnetic resonance imaging indications.

Indication	Description
Any pediatric spine trauma^[26]^	Lower threshold for CCJ injuries among this individuals
Presumption of myelopathy^[27]^	Limb weakness, numbness, tingling, and exaggerated deep tendon reflexes
Neck pain/radiculopathy ^[28]^	Persistent nonspecific symptoms despite conservative management
Cord compression Signs^[27]^	Ruling out epidural collections and post-traumatic annular disc tears

CCJ = craniocervical junction.

Although performing a cervical MRI to exclude ligamentous injuries is challenging due to the time-constraint and relatively long scan duration, missed CCJ injuries can result in severe long-term disabilities and lifelong patient suffering. This emphasizes the importance of enhancing the diagnostic protocols and raising clinical awareness.

## 4. Conclusion

Our case report provides imaging illustration of devastating long-term consequences of an overlooked CCJ injury and review the wide clinical indications for CCJ-MRI in individuals with cervical trauma.

## Author contributions

**Conceptualization:** Mahyar Daskareh, Elham Rahmanipour.

**Data curation:** Mahyar Daskareh, Mohammad Ghorbani.

**Formal analysis:** Mohammad Ghorbani.

**Investigation:** Mahyar Daskareh, Elham Rahmanipour.

**Methodology:** Saeid Esmaeilian, Elham Rahmanipour.

**Project administration:** Mahyar Daskareh, Elham Rahmanipour.

**Resources:** Elham Rahmanipour.

**Supervision:** Mahyar Daskareh.

**Validation:** Saeid Esmaeilian.

**Visualization:** Saeid Esmaeilian.

**Writing – original draft:** Mahyar Daskareh, Saeid Esmaeilian, Mohammad Ghorbani.

**Writing – review & editing:** Mahyar Daskareh, Saeid Esmaeilian, Elham Rahmanipour, Mohammad Ghorbani.
